# Protective Efficacy of Newcastle Disease Virus Expressing Soluble Trimeric Hemagglutinin against Highly Pathogenic H5N1 Influenza in Chickens and Mice

**DOI:** 10.1371/journal.pone.0044447

**Published:** 2012-08-28

**Authors:** Lisette A. H. M. Cornelissen, Olav S. de Leeuw, Mirriam G. Tacken, Heleen C. Klos, Robert P. de Vries, Els A. de Boer-Luijtze, Diana J. van Zoelen-Bos, Alan Rigter, Peter J. M. Rottier, Rob J. M. Moormann, Cornelis A. M. de Haan

**Affiliations:** 1 Central Veterinary Institute of Wageningen UR, Lelystad, The Netherlands; 2 Virology Division, Department of Infectious Diseases & Immunology, Faculty of Veterinary Medicine, Utrecht University, Utrecht, The Netherlands; Virginia Polytechnic Institute and State University, United States of America

## Abstract

**Background:**

Highly pathogenic avian influenza virus (HPAIV) causes a highly contagious often fatal disease in poultry, resulting in significant economic losses in the poultry industry. HPAIV H5N1 also poses a major public health threat as it can be transmitted directly from infected poultry to humans. One effective way to combat avian influenza with pandemic potential is through the vaccination of poultry. Several live vaccines based on attenuated Newcastle disease virus (NDV) that express influenza hemagglutinin (HA) have been developed to protect chickens or mammalian species against HPAIV. However, the zoonotic potential of NDV raises safety concerns regarding the use of live NDV recombinants, as the incorporation of a heterologous attachment protein may result in the generation of NDV with altered tropism and/or pathogenicity.

**Methodology/Principal Findings:**

In the present study we generated recombinant NDVs expressing either full length, membrane-anchored HA of the H5 subtype (NDV-H5) or a soluble trimeric form thereof (NDV-sH5^3^). A single intramuscular immunization with NDV-sH5^3^ or NDV-H5 fully protected chickens against disease after a lethal challenge with H5N1 and reduced levels of virus shedding in tracheal and cloacal swabs. NDV-sH5^3^ was less protective than NDV-H5 (50% vs 80% protection) when administered via the respiratory tract. The NDV-sH5^3^ was ineffective in mice, regardless of whether administered oculonasally or intramuscularly. In this species, NDV-H5 induced protective immunity against HPAIV H5N1, but only after oculonasal administration, despite the poor H5-specific serum antibody response it elicited.

**Conclusions/Significance:**

Although NDV expressing membrane anchored H5 in general provided better protection than its counterpart expressing soluble H5, chickens could be fully protected against a lethal challenge with H5N1 by using the latter NDV vector. This study thus provides proof of concept for the use of recombinant vector vaccines expressing a soluble form of a heterologous viral membrane protein. Such vectors may be advantageous as they preclude the incorporation of heterologous membrane proteins into the viral vector particles.

## Introduction

Highly pathogenic (HP) avian influenza viruses (AIVs) are the cause of bird flu (also called fowl plague), a highly contagious and often fatal disease of poultry. The HPAIV of the H5N1 subtype that emerged in Southern China in 1996 has continued to circulate in domestic poultry to date. In addition, there are concerns about the pandemic potential of H5N1 HPAIV, as hundreds of people have been infected, of which approximately 60% succumbed to the infection. One effective way to combat avian influenza with pandemic potential is through the vaccination of poultry. Conventional, inactivated influenza vaccines must be administered individually and often require a boost vaccination to induce protective immunity. The administration of these vaccines is thus not only time-consuming, laborious and expensive, but may also increase the risk of exposure of people involved to circulating influenza virus. Live vaccines, which elicit both humoral and cellular immune responses, in general provide rapid protection after a single dose. Another advantage of live vaccines is that they do not need to be adjuvant-formulated, thereby limiting production costs. Because the use of live influenza vaccines has obvious safety restrictions, alternative vaccines based on different heterologous viral vectors have been developed, including pox virus [1], adenovirus [2], infectious laryngotracheitis virus [3], baculovirus [4] and Newcastle disease virus (NDV) [5]–[11].

NDV is an enveloped virus with a negative-strand, nonsegmented RNA genome that belongs to the *Paramyxoviridae* family [12]. It is an important pathogen of poultry sometimes causing high rates of mortality depending on the virulence of the infecting strain. Live attenuated, lentogenic NDV strains are used in many countries for vaccination of commercial poultry against infection with virulent, velogenic NDV. The use of lentogenic NDV vectors that express AIV hemagglutinin (HA) genes may be an attractive approach to vaccinate against influenza, as antibodies against HA protect against AIV infection and disease. Indeed, an increasing number of studies has shown that NDV can be successfully used as a vaccine vector for delivery of HA antigen to protect chickens [7], [8] and mammals [5], [6] against influenza. In poultry, such a vaccine does not only protect against influenza virus but also against NDV infection [5], [9]–[11]. Furthermore, it allows serological differentiation of infected from vaccinated animals (DIVA principle) [11], [13].

NDV has a wide tissue/host tropism and can infect various mammalian species in addition to birds [14]. While this characteristic makes NDV a suitable vaccine vector for use in other animals than birds, also humans are among the many species that can be infected with NDV. Infection with NDV is seen most often in people who have been in contact with high doses of virus, as for instance members of vaccination teams performing vaccination with NDV aerosols. Infected people manifest signs of conjunctivitis for a few days and are protected on re-exposure [15]. However, the zoonotic potential of NDV has raised safety concerns regarding the use of live NDV recombinants as vector vaccines. Furthermore, insertion of genes encoding heterologous viral attachment/fusion proteins into the NDV genome, may result in the incorporation of these proteins into the viral envelope and hence in the generation of NDV with altered tropism and/or pathogenicity. Full length HA protein expressed by NDV was indeed shown to be incorporated into NDV particles [7], [11], [16], although a change in tropism or increased virulence was not observed [16]. Although expressed foreign proteins in nonsegmented negative-strand RNA virus in general do not appear to enhance the virulence of the vector or shift its tropism [17], [18], one must confirm this for each vector/insert combination, using both avian and mammalian experimental species [19]. As such safety issues cannot be completely ruled out, the design of alternative approaches, in which NDV is used as a vector vaccine, is warranted.

In a previous study, we demonstrated the vaccine potential of recombinant soluble trimeric subtype 5 hemagglutinin (sH5^3^) produced in mammalian cells [20]. The sH5^3^, which lacks a transmembrane anchor and is therefore secreted from cells, is biologically active as demonstrated by its binding to sialic acid receptors. A single intramuscular vaccination with adjuvanted sH5^3^ antigen conferred complete protection to chickens against a lethal challenge with HPAIV H5N1, whereas mice required a boost immunization for complete protection. The efficacy of the recombinant soluble HA vaccination approach has also been demonstrated using other HA subtypes and different animal models [21]–[23].

In the present study, we modified the genome of NDV strain Herts/33 to generate NDV-sH5^3^, an attenuated NDV vector expressing the soluble trimeric form of H5. As this protein lacks a transmembrane domain, it is not anchored into cellular membranes and hence no NDV virions carrying HA proteins are produced. This approach therefore diminishes the risk of generating a recombinant NDV with altered tropism or increased pathogenicity, resulting from incorporation of heterologous attachment proteins into virions. We examined the ability of this recombinant NDV to induce protection against a lethal H5N1 infection in chickens and mice in comparison to a recombinant NDV expressing the full length H5 protein (NDV-H5). Vaccination of chickens with NDV-sH5^3^ by the intramuscular (IM) route was shown to provide complete protection against a lethal challenge with HPAIV H5N1. However, mucosal immunization reduced vaccine efficacy in comparison with the IM route. The NDV-sH5^3^ vaccine appeared to be ineffective in mice, although in this species the NDV-H5 induced protective immunity after combined ocular and intranasal administration.

## Materials and Methods

### Cells and viruses

Quail muscle (QM5) cells [24] were cultured in QT35 complete medium (Life Technology), supplemented with 5% fetal bovine serum (FBS) and antibiotics. NDV strain Herts/33 was propagated by inoculation into the allantoic cavity of 9 to 11-day-old embryonated specific pathogen-free (SPF) chicken eggs. Allantoic fluid was harvested after 3 days, clarified and aliquoted. Recombinant fowlpox virus fpEFLT7 (herein called FPV-T7), providing stable expression of bacteriophage T7 RNA polymerase in both avian and mammalian cells [25], was propagated in primary chicken embryo liver cells (prepared in the laboratory of the Central Veterinary Institute). HPAIV A/Vietnam/1194/04 H5N1 (kindly provided by Dr. Alan Hay from the WHO Influenza Centre at the National Institute for Medical Research, London) was propagated in 9 to 11-day-old embryonated SPF chicken eggs to produce a virus stock. All experiments with HPAIV, including animal studies, were conducted under BSL3 conditions.

### Construction of NDV cDNAs expressing the HA protein of H5N1 influenza virus

The construction of full-length cDNA of NDV Herts/33, pFL-Herts^AF^, in which the F gene is flanked by the unique restriction sites *Asc*I and *Fse*I, has been described before [26]. In order to generate an attenuated NDV, the polybasic cleavage site of the fusion (F) protein was modified into a monobasic cleavage motif (GRQGR↓L; creating FΔPBC). To this end, the F gene from pFL-Herts^AF^ was amplified by overlap extension (OE)-PCR using primer pair pAscI(Herts) (5′-TTGGCGCGCCCCAGGTGCAAGATGGGC-3′; *Asc*I underlined) and N043 (5′-TTACTAGTTTACGAAAAGTATTGGATTTGTGCCCC-3′), and N044 (5′-GGAGGGAGACAGGGACGCCTTATAGGTGCCATTATCG-3′) and pFseI(Herts) (5′-CCCGATTGAGGGCCGGCCTCCCC-3′; *Fse*I underlined). Both PCR products were added as template in a second PCR using only the flanking primers pAscI(Herts) and pFseI(Herts) to fuse both fragments. The obtained PCR product was cloned between the *Asc*I and *Fse*I site of pFL-Herts^AF^, generating pFL-HertsΔPBC. For insertion of the H5 genes between the P and M genes, *Xba*I and *Pme*I restriction sites were created in the P-M intergenic region of pFL-HertsΔPBC by OE-PCR. First, cDNA of the pFL-HertsΔPBC was amplified with primer pair pMT7 (5′-GTGCTAACGTTTCTTCTCTAAGTGATC-3′; *Acl*I underlined) and pMT6 5′-GTTTAAAGCGAGTTGCGCGATCATCTAGAGGGGTAG-3′; *Xba*I site underlined) and primer pair pMT5 (5′-TCTAGATGATCGCGCAACTGCGTTTAAACTAGCAGC-3′; *Pme*I underlined) and pMT8 (5′-CACTTCCTAGGCAGAGCATCGCAGAGG-3′; *Avr*II underlined). The overlapping PCR products were then fused by PCR performed with the flanking primers pMT7 and pMT8. The resulting PCR fragment was cloned between the *Acl*I and *Avr*II site of pFL-HertsΔPBC. Subsequently, a synthetic linker (5′-GCTCTAGA *TAAGAAAAAATACGGGTAGAA* GTTTAAACGGCGCGCCGG-3′) containing the sequence of the NDV transcription termination and start signals (italics) and *Xba*I and *Pme*I sites (underlined) was inserted using the *Xba*I and *Pme*I sites, resulting in pFL-Herts^XP+^ΔPBC.

The H5N1 A/Vietnam/1194/2004 virus (GenBank accession no. ABW90137.1), lacking a multibasic cleavage site in the H5 protein, was used as the source of the HA gene. Viral RNA was extracted from the virus using a High Pure Viral RNA kit (Roche) and reverse transcribed using Superscript II (Invitrogen) and primer 5′-AGCAAAAGCAGG-3′, matching the non-coding region of each segment. The HA gene was amplified with primers 5′-AGCTTTGTTTAAACA**ATG**GAGAAAATAGTGCTTC TTTTTGC-3′ (start codon in bold) and 5′-CCGGCGCGCCGTTTAAAC **TTA**AAT GCAAATTCTGCATTGTAAC-3′ (stop codon in bold) each containing a *Pme*I site (underlined). The PCR product was digested with *Pme*I and inserted into pFL-Herts^XP+^ΔPBC constructed in the previous step using the created *Pme*I site, resulting in pFL-HertsΔPBC-H5.

To create pFL-HertsΔPBC-sH5^3^, the pCD5-sH5^3^ expression vector was used [20]. This vector encodes the predicted HA ectodomain from A/Vietnam/1194/2004 under the control of a CMV promoter. The H5 sequence is preceded by a CD5 signal peptide-encoding sequence and followed by sequences coding for the GCN4 isoleucine-zipper trimerization motif [27] and the Strep-Tag II (IBA, Germany). In addition, this construct contains *Pme*I restriction sites upstream of the signal peptide and downstream of the Strep-Tag II coding sequences. The pCD5-sH5^3^ plasmid was digested with *Pme*I and the liberated insert was ligated into similarly digested pFL-Herts^XP+^ΔPBC, resulting in pFL-HertsΔPBC-sH5^3^. Each pFL-Herts construct was generated as such that the size of the genome complied with “the rule of six” [28], [29]. All plasmids were cloned according to standard procedures and sequence analysis was performed to verify that no inadvertent changes had been introduced.

### Rescue of recombinant NDV

Recombinant NDV was generated by reverse genetics using the FPV-T7 RNA polymerase system essentially as described previously [30]. Briefly, QM5 cells grown to subconfluency in 6-well dishes were infected with FPV-T7 and transfected with the full-length cDNA and helper plasmids expressing the NDV replication proteins NP, P and L. The supernatant was harvested after 6 days of incubation at 37°C and inoculated (0.2 ml) into the allantoic cavity of embryonated SPF chicken eggs. After incubation for 3 days, the allantoic fluid was clarified, diluted in PBS (1∶1000) and filtered through a 0.2 μm pore filter to remove FPV-T7. The filtrate was then inoculated into the allantoic cavity of embryonated SPF chicken eggs to amplify the virus. Allantoic fluid was harvested after 3 days, clarified and aliquoted. Virus recovered from pFL-HertsΔPBC-H5 was designated NDV-H5 and virus recovered from pFL-HertsΔPBC-sH5^3^ was designated NDV-sH5^3^. Virus titers (TCID_50_ ml^−1^) of the harvests were determined in QM5 cells by the end-point dilution method reading CPE at 5 days post infection (p.i.). and calculated according to Reed and Münch [31].

### Intracellular immunoperoxidase staining and western blot analysis

Expression of the HA protein in QM5 cells after infection with recombinant NDV was analyzed by intracellular immunoperoxidase staining as described [32]. For this, the cells were permeabilized, fixed with 4% paraformaldehyde and incubated with monoclonal antibody (mAb) 15A6 anti-H5 (1∶500; Santa Cruz). Anti-F mAb 8E12A8C3 (1∶5000; CVI, Lelystad) was used for detection of the F protein as control for NDV replication. Horse radish peroxidase-conjugated rabbit-anti-mouse immunoglobulin (1∶1500; Dako P0260) was used as the secondary antibody. Binding of this antibody was detected using 3-amino-9-ethylcarbazole (AEC) peroxidase substrate (Sigma), enzymatic conversion of which produces a red reaction product. HA protein expression by recombinant NDV was confirmed by sodium dodecylsulfate (SDS)-polyacrylamide gel electrophoresis (PAGE) followed by western blotting. At 64 h p.i., combined lysates were prepared of the cell culture media and cells using Passive Lysis Buffer (Promega). Proteins in the lysates were separated by SDS-PAGE, transferred onto an Invitrolon™ PVDF filter (Invitrogen) and probed with the anti-H5 mAb. Binding of the second peroxidase-conjugated anti-mouse antibody (Dako P0260) was visualized by chemiluminescence using the Supersignal kit (Pierce).

### Production and purification of sH5^3^

For the production of recombinant sH5^3^ protein, QM5 cells were infected with NDV-sH5^3^ diluted in trypsin-free DMEM at a multiplicity of infection (moi) of 3 TCID_50_ units per cell. Tissue culture supernatants were collected (10 ml) after 72 h of incubation at 37°C and subsequently clarified by low-speed centrifugation. The pH of the harvest was adjusted to 7.8 and Avidin (IBA, Germany) was added until a final concentration of 3 μg ml^−1^. After 4 h, recombinant sH5^3^ protein was purified using Strep-Tactin sepharose beads as described by the manufacturer (IBA, Germany). The sH5^3^ protein concentration was determined with a nano-drop spectrophotometer and estimated on gel using a series of known amounts of bovine serum albumin (BSA) as reference. To this end, the purified sH5^3^ and the BSA samples were subjected to SDS-PAGE after which proteins were stained with Blue stain reagent (Thermo Scientific). The oligomerization status of the sH5^3^ protein was determined by blue native-PAGE analysis followed by western blotting as described above using a mAb anti-Strep-tag (IBA, Germany).

### Intracerebral pathogenicity index

All animal experiments were conducted in the high containment facility of the Central Veterinary Institute. The standardized chicken pathogenicity test was performed as described in the European Community Council Directive 92/66/EEC [33]. Briefly, 10 one-day-old SPF chickens (Lohmann, Germany) were injected intracerebrally with 0.05 ml of a 1/10 dilution of the egg-grown HertsΔPBC virus and observed for clinical signs every day over a period of 8 days. The birds received a score of 0 if they appeared normal, 1 if they were sick, and 2 if they were dead. The intracerebral pathogenicity index (ICPI) was calculated as the mean score per bird per observation.

### Vaccination experiment in chickens

SPF White Leghorn chickens (Lohmann, Germany) of 6 weeks old were used in this experiment. Groups of 10 chickens were vaccinated with 10^7^ TCID_50_ of vaccine virus either by the IM (0.5 ml) or the combined oculonasal/ intratracheal (ON/IT; 0.2 ml) route. In addition, one group of 10 chickens was vaccinated IM with 5 µg of recombinant sH5^3^ protein adjuvanted with Stimune (Prionics, [34]). Another group of ten chickens received PBS ON/IT as well as PBS in Stimune IM to serve as mock-vaccinated control for each administration route. The experimental groups were housed in separate rooms. In each group, two sentinels were housed together with the test animals (from day one p.v. onwards). All birds were challenged 3 weeks post vaccination (p.v.) by intranasal (0.1 ml) and intratracheal (0.1 ml) inoculation of in total 10^5^ TCID_50_ of H5N1 HPAIV A/Vietnam/1194/04. The chickens were daily inspected for signs of disease during the entire experimental period. Observations of clinical signs and mortality post challenge (p.c.) were recorded for 10 days according to the following scoring system: 0 =  healthy, 1 =  sick, 2 =  severely ill, and 3 = dead. Chickens were inspected twice a day as long as severe illness (score of 2) was scored. Birds that were reluctant to move in response to manipulation or unable to reach the feeding and water bin were euthanized. The clinical index was calculated as the mean score per bird per observation. Blood samples were collected at 3 weeks after immunization. Tracheal and cloacal swabs were taken on day 2, 4 and 7 p.c in order to assess shedding of challenge virus.

### Vaccination experiment in mice

Female, 7-week-old BALB/c mice (SPF; Charles River Laboratories, Germany) were allocated to 5 experimental groups with 10 animals each. For vaccination, mice were anaesthetized with ketamin/xylazine intraperitoneally and inoculated oculonasally (ON; 50 μl) or IM (50 μl in each hind leg) with 10^6.5^ TCID_50_ of vaccine virus. As challenge control, one group of mice received PBS ON (50 μl) and PBS in Stimune IM (100 μl). Three weeks after vaccination, mice were anaesthetized as described above and infected intranasally (50 μl) with 10 times the 50% lethal dose (LD_50_) of the homologous H5N1 A/Vietnam/1194/04 virus. Mice were weighed and observed daily for signs of disease for the next 14 days. Clinical signs were scored on a scale of 0 to 4, wherein 0 =  no clinical signs; 1 =  rough coat; 2 =  rough coat, less reactive, passive during handling; 3 =  rough coat, rolled up, laboured breathing, passive during handling; 4 =  rough coat, rolled up, laboured breathing, inactive in response to manipulation/ handlings. Mice were inspected twice a day as long as they received a score of 3 and were euthanized when they received a score of 4. Blood samples were collected at 3 weeks after the immunization. Surviving animals were bled and sacrificed on day 14 p.c.

### Ethics statement

All animal studies were performed in accordance with the Dutch Law on Animal Experiments (Wod, ID number BWBR0003081). The study protocols were approved by the Animal Ethics Committee of the Central Veterinary Institute of the Wageningen UR (Permit Numbers: 2009011, 2010006 and 2010119). Chicken embryos (14 days of age) used for the collection of liver tissue were humanely euthanized by decapitation. Embryonated eggs used in the propagation of virus were cooled at 4°C for at least 4 h prior to harvest of allantoic fluid.

### Viral RNA detection by real time RT-PCR

Viral RNA was extracted from 0.2 ml of pooled tracheal and cloacal swabs collected from each bird on the particular sampling days. The amount of viral RNA was analyzed by real-time RT-PCR amplifying the M1 gene of influenza A virus [35] similarly as described before [20], this time using a Mx3005p thermocycler (Agilent Technologies). Viral RNA present in serial 10-fold dilutions of H5N1 A/Vietnam/1194/04 with known virus titer was analyzed in parallel to generate a standard curve for correlation of cycle threshold (*C_T_*) values with virus infectivity titers. As control for cross-over contamination, negative samples (PBS) were included at random positions on the test plates.

### Serum antibody detection

Heat-inactivated immune sera from chicken blood samples were tested in two-fold serial dilutions for hemagglutination inhibition (HI) activity with 1% chicken red blood cells and 4 hemagglutinating units (HAU) of the particular virus. Immune sera prepared from mouse blood samples were treated O/N with Vibrio Cholera derived neuraminidase (Roche), heat-inactivated at 56°C for 30 min and tested by HI assay using turkey red blood cells as described above. Antibody titers were expressed as the reciprocal of the highest serum dilution showing HI.

## Results

### Generation of NDV-H5 and NDV-sH5^3^ recombinant viruses and expression of HA proteins

The cleavage site of the NDV F protein is an important determinant of virulence in chickens [30], [36]. Velogenic strains, including Herts/33, typically contain F proteins with multibasic cleavage sites, which are cleaved by furin-like proteases, and have an ICPI of 1.2 to 2.0. In contrast, lentogenic viruses contain F proteins with a single basic amino acid and have an ICPI of 0.7 or less [14], [36]. In order to generate an attenuated virus, the full-length cDNA clone of Herts/33 was modified such that the encoded F protein had a monobasic cleavage motif (pFL-HertsΔPBC). Intracerebral inoculation with virus recovered from pFL-HertsΔPBC resulted in an ICPI of 0.04, indicating that this virus is indeed low-pathogenic to chickens.

**Figure 1:**
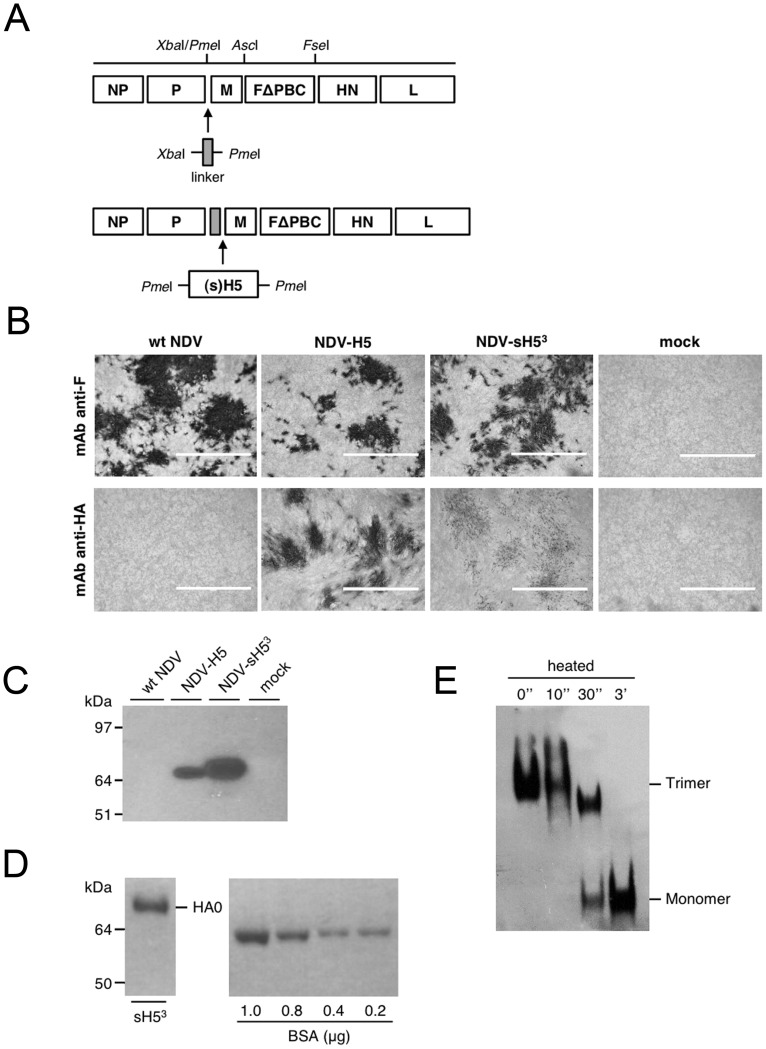
Construction of recombinant NDV, expression and purification of H5 protein. A) Construction of pFL-HertsΔPBC expressing influenza H5N1 HA proteins. The sequence of the full-lenght cDNA of Herts/33, which codes for the multibasic cleavage site of the F protein was mutated into a monobasic cleavage site-encoding sequence (FΔPBC) resulting in pFL-HertsΔPBC. The HA gene of H5N1 A/Vietnam/1194/04, encoding either the full length or the soluble trimeric form, was cloned between the *Pme*I sites created in the intergenic region of the P and M genes of the pFL-HertsΔPBC vector following insertion of a linker containing NDV transcription termination and start signals. NP, nucleoprotein; P, phosphoprotein; M, membrane protein; F, fusion protein; HN, hemagglutinin-neuraminidase; L, large polymerase. B) Expression of HA protein by recombinant NDV in QM5 cells. Cells were infected with wt NDV (Herts/33), NDV-H5, NDV-sH5^3^ or were mock-infected and analyzed by immunocytochemistry using mAb specific for NDV F or influenza virus H5. Scale bars represent 400 μm. C) Western blot analysis with an anti-H5 mAb following SDS-PAGE of combined lysate of QM5 cells and the cell culture supernatant (20 μl/T75 flask). The MW of the marker proteins is indicated on the left. D) The sH5^3^ protein was purified from the supernatant of NDV-sH5^3^-infected QM5 cells and subjected to SDS-PAGE (∼ 1 μg/15 μl), after which proteins were stained with Blue stain reagent. A series of BSA dilutions were run in parallel. E) Blue native PAGE analysis of sH5^3^ purified from the cell culture supernatant of NDV-sH5^3^-infected QM5 cells (∼ 1.7 μg/20 μl). The position in the gel of the trimeric and monomeric form of the H5 protein is indicated. Prior to gel electrophoresis some samples were heated at 95°C for the indicated time periods.

Next, we generated two NDV recombinant viruses, NDV-H5 and NDV-sH5^3^, expressing either the full length HA (H5) or a soluble trimeric form (sH5^3^) of the HA protein, respectively. Previously Wei and coworkers [37] have shown that soluble trimeric HA proteins are superior antigens when compared to their monomeric counterparts. The H5 encoding sequences were each inserted between the P and M genes of the pFL-HertsΔPBC vector (Fig. 1A). For proper expression, the HA genes were flanked by NDV transcription start and termination signals. The recombinant NDV-H5 and NDV-sH5^3^ could be readily recovered after transfection of the full-length constructs into QM5 cells. Both viruses replicated to high titers in embryonated chicken eggs; NDV-H5 and NDV-sH5^3^ yielded a titer of 8.0 log_10_ and 8.5 log_10_ TCID_50_ ml^−1^, respectively. NDV-H5 and NDV-sH5^3^ both had an ICPI value of 0.00. RT-PCR performed on the allantoic fluid and subsequent sequencing of the PCR products confirmed the presence of the proper H5-encoding inserts in the viral genomes.

To verify expression of the HA proteins during infection, QM5 cells were infected with egg-cultured wild type (wt) NDV(Herts/33), NDV-H5 or NDV-sH5^3^ or they were mock-infected. At day 5 p.i., they were subjected to immunocytochemistry using mAb specific for NDV F- or influenza virus H5. The results show that many cells in the monolayer were infected with wt NDV or the recombinant NDVs, while no F protein could be detected after mock infection (Fig. 1B). In addition, similar numbers of cells were positive for the H5 protein when cells had been infected with NDV-H5 and NDV-sH5^3^. Less intense staining was observed for cells infected with NDV-sH5^3^ relative to the NDV-H5-infected cells, presumably because the sH5^3^ protein does not accumulate in the cells but is secreted into the cell culture medium. No H5-specific staining was observed in cells infected with wt NDV. The HA protein expression levels were further analyzed by subjecting from each infection a sample of the combined QM5 cell lysate and cell culture supernatant to SDS-PAGE analysis followed by western blot using an anti-H5 mAb. As expected, proteins with an apparent molecular weight of ∼70 kDa, corresponding to either the uncleaved full length H5 protein or the soluble H5 protein containing the trimerization motif, were detected after infection with the recombinant NDVs but not after infection with the wt NDV (Fig. 1C). It appeared that the sH5^3^ protein reached a ∼3 fold higher expression level than its full length counterpart. The recombinant sH5^3^ protein was purified from the supernatant of NDV-sH5^3^-infected QM5 cells by making use of the StrepTag. Blue stain SDS-PAGE analysis of the purification product again revealed a ∼70 kDa protein with an estimated yield of 0.9 mg per 100 ml of cell culture medium based on a series of known amounts of BSA that were run in the same gel (Fig. 1D). To confirm its oligomeric nature, the purified sH5^3^ protein was subjected to blue-native gel electrophoresis (Fig. 1E). When the H5 preparation was heat-denatured for increasing time periods prior to electrophoresis, the initially oligomeric HA species, presumably corresponding to trimers based on previous results [20], dissociated into monomers. In conclusion, we generated recombinant attenuated NDVs that express either the full length or soluble trimeric H5 protein.

### Protective efficacy of NDV-HA, NDV-sH5^3^ and sH5^3^ protein against lethal HPAIV H5N1 infection in chickens

**Figure 2:**
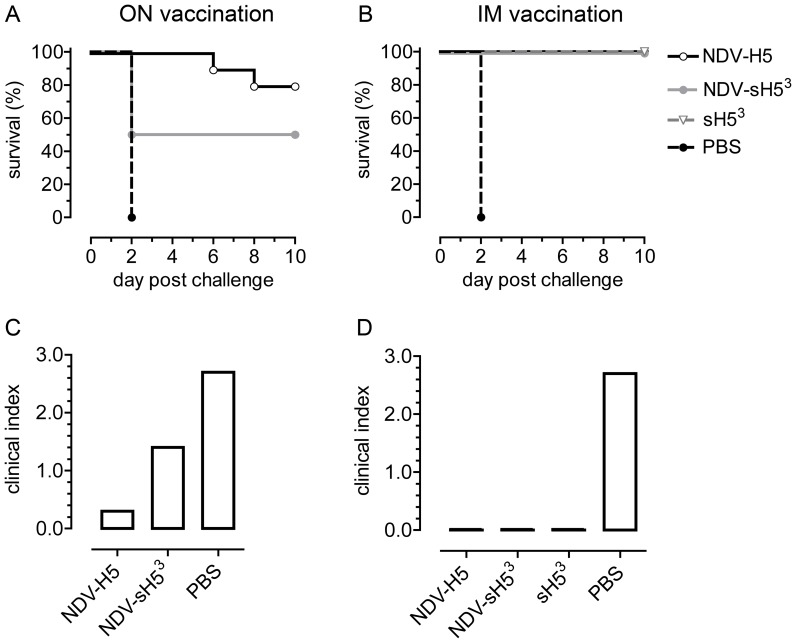
Vaccination of chickens. Groups of 10 SPF chickens were immunized with 10^7^ TCID_50_ of NDV-H5 or NDV-sH5^3^, either via the ON/IT or IM route. Another group received an IM immunization with sH5^3^ adjuvanted in Stimune. As a challenge control, one group of chickens was mock-vaccinated with PBS. Three weeks after the vaccination, birds were challenged with ∼10^5^ TCID_50_ of HPAIV H5N1. A and B) Kaplan-Meier survival curves indicating percentage of survival p.c. on each day for each group that was (mock-)vaccinated via the ON/IT (A) or IM (B) route. C and D) Clinical index calculated on basis of clinical signs (as described in materials and methods) observed after challenge for each group that was (mock-)vaccinated ON/IT (C) or IM (D). An index of 3.0 means that all birds died within 24 hours. For reference, each panel includes the same PBS group.

To examine the protective efficacy of NDV-sH5^3^ compared to NDV-H5, groups of 10 chickens were vaccinated once with a dose of 10^7^ TCID_50_, either via the ON/IT or IM route. Three weeks later, the chickens were challenged by inoculation with a lethal dose of HPAIV H5N1. In parallel, as a control, one group of chickens was vaccinated IM with Stimune-adjuvanted sH5^3^ that had been purified from the cell culture supernatant of NDV-sH5^3^-infected QM5 cells (Fig. 1D). As a challenge control, one group of chickens received PBS. None of the chickens showed symptoms of disease over the 3-week observation period after vaccination, in agreement with the ICPI values determined for the recombinant NDVs lacking the multibasic cleavage site (see above). The percentages of chickens surviving the challenge and the mean clinical scores per group after the challenge are shown in Fig. 2. All mock-vaccinated birds died from the challenge within 2 days (Fig 2A), resulting in a clinical index of 2.7 out of a possible maximum of 3.0 (Fig. 2B). Five out of 10 chickens that received NDV-H5^3^ via the ON/IT route died within 2 days; of the 5 survivors, however, only one showed illness (a score of 1 between day 7 and 10), resulting in an overall clinical index of 1.4. ON/IT delivery of NDV-H5 induced sufficient immunity to protect 80% of the chickens (Fig. 2A), with one animal dying on day 6 and 8 each. None of the survivors showed clinical signs, resulting in a clinical index of 0.3 (Fig. 2B). Vaccination of animals via the IM route with NDV-sH5^3^ provided complete protection; all chickens survived without showing symptoms indicative of influenza-related disease (Fig. 2C and D). Similar results were obtained when animals were vaccinated IM with NDV-H5 or with adjuvanted sH5^3^.

**Figure 3:**
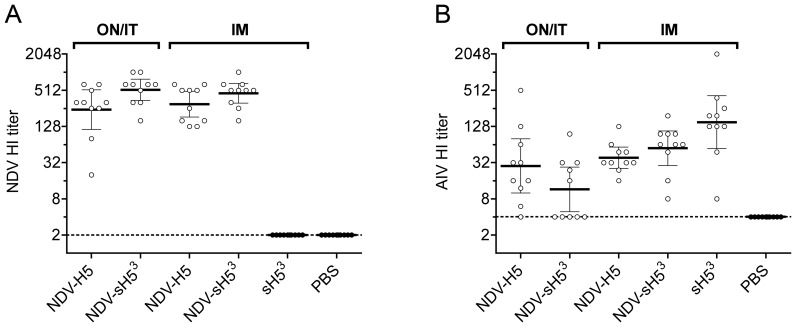
Serum antibody responses in vaccinated chickens. HI antibody titers against A) NDV Herts/33 or B) AIV measured in individual serum samples collected on day 21 p.v. via ON/IT or IM route. Titres are expressed as the reciprocal of the highest serum dilution showing HI. The dashed line indicates the lower limit of detection. Titers below the detection limit were assigned a value of 2 or 4, respectively for calculation of the mean. Horizontal bars represent the geometric mean titers of the groups and vertical bars represent 95% confidence intervals. Serum titers from chickens that died after the challenge are indicated by filled circles, the open circles correspond to animals that survived the challenge. For reference, each panel includes the same PBS group.

Antibody responses against NDV and AIV were measured in individual serum samples collected immediately prior to challenge (day 21) by HI assays. Effective induction of NDV vector-specific HI antibodies was observed after immunization with NDV-H5 and NDV-sH5^3^ (geometric means ranging between 243 and 521) regardless of the route of administration used (Fig. 3A). Mock-vaccinated chickens and chickens that received sH5^3^ protein all had a NDV-specific HI titer below the detection limit and were assigned a value of 2, although these latter, but not the former, animals clearly displayed AIV-specific HI titers (Fig. 3B). Following IM vaccination, AIV-specific HI antibody titers in chickens that received NDV-sH5^3^ ranged from a maximum of 192 to a minimum of 8 (mean titer of 55.4), comparable to those detected in NDV-H5 vaccinated birds (mean titer of 38.7; Fig. 3B), which was apparently sufficient to protect these birds against the lethal challenge. Essentially the same antibody titers (mean titer of 28.2) could be detected after ON/IT vaccination with NDV-H5, except in two chickens, both of which had a titer of 6 or lower and did not survive the challenge. After ON/IT vaccination of animals with NDV-sH5^3^, chickens that survived the challenge had developed antibody levels within the same range as the other protected animals (96-16), while no antibodies were measurable in the chickens that died, resulting in a mean titer of 11.5. The results show that survival of vaccinated chickens corresponds with the induction of HI titers of 8 or higher. While all animals vaccinated with the recombinant NDVs reached protective HI titers after IM immunization, ON/IT administration was less effective, which was most apparent for the animals that received NDV-sH5^3^. As no NDV- or AIV-specific HI titers were detected after IM immunization with UV-inactivated NDV, we conclude that replication of NDV was required for the observed immunogenicity (data not shown).

### Shedding of challenge virus

To examine whether vaccination with recombinant NDVs protected chickens from shedding challenge virus, tracheal and cloacal swabs sampled from each chicken on day 2, 4 and 7 p.c. were pooled and tested for the presence of viral RNA using a quantitative RT-PCR assay detecting the M1 gene. The results are shown in Table 1. All chickens that were immunized with NDV vector vaccine tested positive at day 2 and/or day 4 p.c., at least when they survived the challenge for that time period. The birds, vaccinated with recombinant NDV vector vaccine, that were protected against the lethal challenge did not shed challenge virus at day 7 p.c., i.e. no viral RNA could be detected in the swabs taken from these birds at that time point. Of all the birds that received sH5^3^, only two tested positive for viral RNA in their swabs and only on day 2 p.c. Notably, these two birds developed the lowest AIV-specific antibody titers of the group (Fig. 3B). In conclusion, birds vaccinated with NDV vector vaccine no longer shed virus at day 7 p.c., although vaccination with the adjuvanted recombinant protein preparation appeared to be more effective in diminishing virus shedding.

**Table 1:** Detection of H5N1 challenge virus in tracheal and cloacal swabs of vaccinated chickens.

	ON/IT vaccination*^a^*						IM vaccination								
	NDV-H5			NDV-sH5^3^			NDV-H5			NDV-sH5^3^			sH5^3^		
Bird #	D2	D4	D7	D2	D4	D7	D2	D4	D7	D2	D4	D7	D2	D4	D7
1	2.3	−	−	1.9	−	−	3.6	−	−	5.0	−	−	−	−	−
2	−	2.5	−	x	x	x	3.9	2.7	−	3.1	−	−	−	−	−
3	3.9	5.7	x	2.3	−	−	4.1	−	−	−	4.6	−	−	−	−
4	1.6	−	−	x	x	x	−	−	−	3.7	2.8	−	−	−	−
5	4.0	4.0	x	x	x	x	3.3	−	−	3.3	−	−	−	−	−
6	2.8	3.5	−	x	x	x	4.6	2.9	−	2.8	−	−	1.6	−	−
7	−	4.4	−	x	x	x	4.2	−	−	−	1.2	−	−	−	−
8	4.4	4.0	−	2.7	−	−	3.2	4.5	−	−	−	−	4.2	−	−
9	−	1.6	−	1.8	−	−	3.5	1.8	−	−	1.8	−	−	−	−
10	2.6	3.4	−	−	−	−	3.6	5.3	−	−	5.8	−	−	−	−

a Day (D) post infection on which tracheal and cloacal swabs were collected are indicated. Amount of RNA detected in swabs is expressed as log10 TCID50 ml-1 equivalents.− =  negative (C_T_ >39.0); x  =  not tested because chicken did not survive after challenge with HPAIV H5N1.

### Protective efficacy of NDV-H5 and NDV-sH5^3^ against lethal HPAIV H5N1 infection in mice

**Figure 4:**
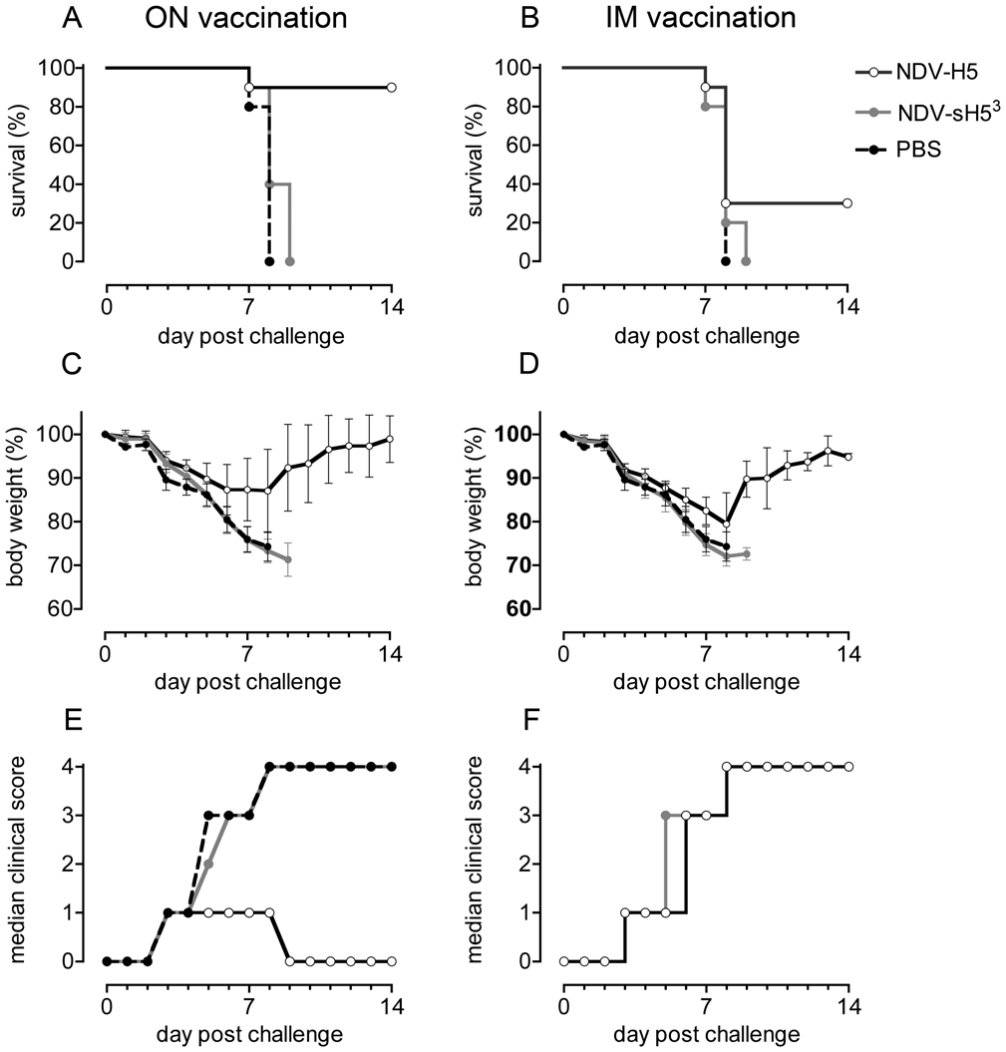
Vaccination of mice. Groups of 10 BALB/c mice were vaccinated with 10^6.5^ TCID_50_ of NDV-H5 or NDV-sH5^3^, either via the ON or IM route. One group of mice was mock-vaccinated (PBS) as challenge control. Three weeks after the vaccination, mice were infected with ∼10 LD_50_ of HPAIV H5N1 and weighed daily and observed for clinical signs during 14 days. Graphed for each group are A and B) Kaplan-Meier survival curves indicating percentage of survival p.c., C and D) mean percentage of body weight changes relative to starting weights measured on the day of challenge (day 0) with error bars representing the standard deviation, and E and F) median clinical scores observed after challenge. For reference, each panel includes the same PBS group.

The efficacy of NDV-sH5^3^ and NDV-H5 vector vaccines was also examined in mice. To this end, groups of 10 mice were vaccinated ON or IM and challenged three weeks later with ∼10 LD_50_ of HPAIV H5N1. The percentage of mice surviving the infection, body weights and median clinical scores per group observed after the challenge are shown in Fig. 4. The mock-vaccinated mice, which all showed respiratory distress and significant weight loss, died at day 7 or 8 p.c. Vaccination with NDV-sH5^3^ did not protect mice from a lethal challenge, regardless of the route of vaccine administration. In both these groups, mice started to lose weight soon after infection, which continued until they died. Intramuscular vaccination with NDV-H5 also did not protect mice against disease and concurrent weight loss; yet 30% of the vaccinated animals survived. These animals lost weight comparable to the control group until day 8 p.c., after which they regained weight. At day 12 p.c. they appeared healthy again. In contrast, ON administration of NDV-H5 conferred protection to 90% of the mice. This group showed only mild signs of disease (a median score of 1) and only a limited decline (∼10%) in body weight. Strikingly, none of these survivors had HI antibodies against NDV or AIV. In conclusion, while NDV-sH5^3^ was unable to protect mice against a lethal challenge with H5N1, protection was observed after ON but not after IM vaccination with NDV-H5.

## Discussion

Once the epidemic of H5N1 HPAIV among poultry was recognized as a potential threat to human health, policy makers, vaccine manufacturers and researchers have joined forces to develop convenient, safe, effective and cost-efficient vaccines against HPAIV for poultry and humans. Because the use of live influenza virus strains has safety restrictions, several alternatives including heterologous live vector viruses expressing the influenza H5 antigen are being explored. The use of recombinant lentogenic NDV as vector vaccine against H5N1 is an attractive approach, as vaccination of poultry with such viruses is expected to result in protection both against infection with velogenic NDV and HPAIV. However, the use of NDV live vector vaccines also raises safety concerns as heterologous viral attachment and fusion proteins may be incorporated into NDV virions as has been demonstrated for HA of AIV [7], [11], [16]. An NDV vector expressing HA in a soluble trimeric form that is released from cells might offer a safe alternative, while retaining the advantages of a live vaccine and of expression of a native HA antigen. In the present study, we generated recombinant attenuated NDV expressing either trimeric sH5^3^ or full length H5 by reverse genetics using the backbone of the Herts strain. The potential of these NDV recombinants as live vaccine was studied by evaluating their ability to protect chickens and mice against a lethal challenge with H5N1 HPAIV. Although the protective efficacy of NDV expressing full length H5 was generally greater than that of NDV expressing sH5^3^, chickens were fully protected against the lethal challenge after a singly immunization with NDV-sH5^3^ when administered via the IM route.

Deletion of the multibasic cleavage site in the F protein of the velogenic NDV strain Herts/33, resulted in an attenuated virus that was subsequently used as a vector for vaccinating chickens against HPAIV. IM or ON/IT immunization with such an attenuated NDV expressing full length H5 protected 100% or 80% of the vaccinated chickens, respectively, against a lethal challenge with HPAIV H5N1. Differences in protection levels correlated with the observed AIV HI titers in the chicken sera. Protected chickens did not shed the challenge virus at day 7 p.c. Previous experiments, in which chickens were vaccinated with NDV-HA recombinants based on attenuated NDV strains, resulted in variable degrees of protection ranging from 40% (immunization with NDV strain Hitchner B1 expressing H7 via eye drop method) [10] to 90% (high-fusogenic NDV vector expressing chimeric F-H7 applied by eye drop) [9] or 100% (ON administration of NDV strain LaSota expressing H5) [5], [11]. There are several factors which could explain the different results, including differences in replication of the NDV strain, HA gene insertion site, HA subtype, administration route, and the vaccine or challenge dose used.

In the present study, chickens developed comparable levels of HI antibody titers against NDV, regardless of the administration route used, indicating that infection and spread of NDV-H5 in the muscular tissue is not a limiting factor for an adequate immune response. Although protection against NDV was not assessed, the mean NDV HI titers were sufficiently high to assume that, with reference to observations by Veits and coworkers [11], the vaccinations would also have protected the chickens against virulent NDV infection.

The protective efficacy of NDV expressing sH5^3^ in chickens proved to be as high as that of NDV expressing full length H5 when given via the IM route: all animals were protected, while similar AIV HI titers were induced. In contrast, NDV-sH5^3^ administered by the ON/IT route provided lower protection and elicited lower AIV HI antibody titers than NDV-H5 delivered via this route (50% vs 80%). The lower antibody levels may be the consequence of a reduced amount of HA antigen exposed to the immune system during NDV-sH5^3^ infection of the respiratory tract, even though lentogenic NDV replicates most efficiently at that site [38]. The soluble H5 proteins may very well be trapped via their efficient binding to the α2-3-linked sialic acid receptors [20], [39] that are omnipresent in mucus and on the epithelial cells lining the respiratory tract of chickens [40]. When trapped by its interaction with sialic acid receptors, sH5^3^ may be less immunogenic than as free trimer or as membrane-anchored, full length HA. A similar difference in protective efficacy was observed after vaccination of mice with our recombinant NDVs. The mouse respiratory tract is also known to be rich in α2-3-linked sialic acid [41], [42], resulting in entrapment of recombinant soluble HA. When this scenario is correct, introduction of point mutations that abolish sialic acid-binding by HA [43] would be expected to increase the protective efficacy of NDV-sH5^3^. Alternatively, it has been suggested that influenza viruses allow efficient cross-linking of B cell receptors because they present HA molecules in a repetitive array [44]. Such arrangement, which will also appear at the membranes of NDV-H5 virions and possibly of cells infected with NDV in the case of NDV-H5 but not of NDV-sH5^3^, may result in a better immune response. Differences in immunogenicity between full length and soluble H5 are not likely to result from differences in glycosylation as both immunogens carry the same number of N-linked glycosylation sites.

While NDV-H5 effectively immunized chickens after administration by the IM route, the mucosal immunization route was clearly superior in mice. After a single ON dose with NDV-H5, 90% of the mice were protected against a lethal infection by HPAIV H5N1, whereas none of the animals were protected after IM administration. Unlike chickens, none of the mice developed detectable levels of HA- or NDV specific antibodies after IM immunization, which may be explained by inefficient replication of NDV after IM administration. However, also after ON vaccination, which resulted in protection of 90% of the mice for NDV-H5, no HA- or NDV-specific antibodies were detectable in serum. As mice vaccinated with NDV-sH5^3^ were not protected at all, an aspecific vector immunization effect can be excluded. Apparently, the absence of serum antibodies does not necessarily imply that the vaccine is ineffective. Likewise, mice [5] and primates [6], which had been vaccinated with a similar NDV-vectored vaccine, displayed very low or no measurable serum HI or neutralizing antibody titers to influenza virus. Protection despite the lack of serum antibodies to AIV suggests that immunity in these mice is mediated by a mucosal rather than a systemic antibody response or antibody-independent (T-cell mediated) immunity, which restrict replication of influenza virus in the upper respiratory tract. It is has been shown that NDV is a very efficient inductor of type I interferons, which promote antigen presentation through the MHC class I pathway mediating activation of CD8+ T-cells [45].

Our study showed, in agreement with other studies, that attenuated NDV expressing full length HA is an attractive live vaccine candidate to protect poultry and possibly also other (mammalian) species against HPAIV. In addition, we investigated the protective efficacy of recombinant NDV expressing soluble trimeric HA proteins, thereby excluding the possibility of HA incorporation into the NDV particle. Although a single administration of NDV-sH5^3^ vaccine by the IM route provided complete protection against a lethal HPAIV H5N1 challenge in chickens and was partially protective when administered ON/IT, this vaccine candidate appeared less effective than NDV expressing full length H5, which was most apparent in mice. Nevertheless, this study provides proof of concept for the use of recombinant vector vaccines expressing a soluble form of a heterologous viral membrane protein. Such vectors may be advantageous as they preclude the incorporation of heterologous membrane proteins into the viral vector particles, which may result in the generation of vaccine vectors with altered tropism and/or pathogenicity. In our view, it will therefore be of interest to study the applicability of this concept also for other vector-immunogen combinations.
